# One Health for Headaches: A Clinical Scientist Residence Project

**DOI:** 10.3390/ijerph20065186

**Published:** 2023-03-15

**Authors:** Paolo Martelletti

**Affiliations:** Department of Clinical and Molecular Medicine, Sapienza University, 00189 Rome, Italy; paolo.martelletti@uniroma1.it

**Keywords:** headaches, inter-multidisciplinary, networking, unmet needs, integration, socioeconomic issues, stakeholders, citizens, stigma

## Abstract

Headaches are non-communicable diseases and have a well-perceived stigma and the greatest personal, biopsychosocial, and occupational burden. The focus of biomedical research has brought attention to certain aspects, such as occupational, educational, and health organization impacts, favoring aspects of therapeutic innovation. These aspects are viable in countries with a high gross domestic product but are less so in countries with a low or average level of development, where dedicated health infrastructures, advanced drugs, and even disease awareness and basic education are lacking. Here, we propose the idea of a One Health project that includes headaches, where the patient is not seen as a single unit but as a high user of public health facilities, a low-efficiency worker, and a citizen with a clear social stigma. This hypothesis of the development of a self-assessment tool is based on seven domains, whose results will be offered for validation and evaluation to stakeholders, scientific societies, research groups, and key opinion leaders, in order to provide a framework of the specific needs per area of intervention (awareness, research, and education, etc.), per geographical area.

## 1. Introduction

After more than three decades of cultural development surrounding headaches, characterized by the use of shared classificatory terminologies, epidemiological evidence, a clear personal and social impact, clear mechanistic visions, revolutionary pharmacological discoveries, and the awareness of widespread multilevel educational structures for health professionals working in this clinical area, more questions arise. Are all the active competencies in this system coordinated and proactive, and are they mutually synergetic and aligned in a single framework? Does the evident multiaxiality of interventions increase operability, or, if not synchronized, does it decrease it? Are the different needs of individual patient populations within different social or national contexts considered to avoid the dispersion of resources and activities, directed towards targets that are not yet sensitized or still at an incomplete level of health development?

A failure to answer these questions could make it difficult to achieve the well-being goals now considered strategic in the United Nations’ The 2030 Agenda for Sustainable Development, which calls for global action to reduce the inequalities of access to health and education, and to improve social policies, as seen in Goal 3, Good Health and Well-Being [1].

Primary headaches account for a considerable share of non-communicable diseases in terms of prevalence, disability, burden, and stigma [2,3,4,5,6,7].

Headache health inequalities and social determinants are not only representative of nations with a low gross domestic product (GDP), but also of advanced nations that, for various economic, organizational, and social reasons, cannot guarantee or are forced to limit access to care for a proportion of individuals with headaches [8,9].

Despite the continuous increase in the average level of headache management capacity observed recently, major inequalities in access to headache care persist within and between geographical macro-areas. The acknowledged health gap between undeveloped and developed countries has repercussions on health systems that struggle to keep up with modern knowledge and the possibilities for educational interventions [10,11,12].

Now, the definition of an intervention framework shared by the field’s international and national stakeholders is needed, and an action plan tailored to these needs and geographical gaps must be proposed.

## 2. Preparedness Plan for Headache

To assess the most effective intervention methods, the development of a headache self-assessment tool must be submitted to the stakeholders involved in headache management in different geographical contexts. This tool should be developed by a small panel of headache experts who, after imagining the process, could develop the domains and interpret the results, thus providing a diversified action plan for various needs. These essential domains are reported in Table 1.

The tool will have to be administered online to the headache area stakeholders, or in the absence of specific scientific societies dedicated to these research groups, to the local key opinion leaders. This will make it possible to highlight indications that are aggregated by geographic areas. Starting with the results of the self-assessment, it will be possible to identify the areas for improvement in the studied settings, and the actions that would strengthen their capacity to prepare for a public health emergency. Only this way would international stakeholders that have more advanced training be able to provide targeted, cost-effective, and tailored help guaranteeing real potential for improvements in headache management. This will help to improve one-shot conferences from hyper-skilled scientists who are able to offer excellent notions and concepts on cultural trips, that, without a prior analysis of the real needs and reception capacities of a specific audience, struggle to guarantee a minimum follow-up evaluation of the long-term usefulness of the generated effects.

## 3. Conclusions

This One Health action plan proposal for headaches is, in part, already inherent and operational in international academic-based headache training schools and various educational programs, which are offered worldwide by the healthcare providers active in headache field. Although these high-performance schools and programs are oriented in clinical education, and in some cases in the preparation of future researchers, they have not yet developed expertise for the transfer of organizational knowledge. This would contribute to a model for building headache-specific healthcare facilities, which could diminish the inequalities in the access to the standard of care of headaches in disadvantaged areas. 

In this sense, proposals that are oriented towards the disability/work capacity ratio already exist in the literature [13,14,15,16], but they must be contextualized in this *One Health for Headache* action plan through the hypothesis of a *Clinical Scientist Residence Program (CSRP)*. This project envisages that, alongside the theoretical phase, a care model design that is compatible with local facilities can be implemented for a short stay. The theoretical phase can benefit from several teaching aids, such as headache book series [15] and management guidelines that can, at different levels of intervention, allow for the use of drugs that are locally available, from the standard of care up to the most recent drugs that target calcitonin-gene-related peptide [17,18,19,20].

This bottom-up CSRP model would produce more beneficial effects by being able to, peripherally, leave an operational model in the field, such as a simple headache clinic, more so than top-down models that, as legacies of brilliant lectures, do not germinate useful activities in the context of everyday reality. The same applies to visiting scientist projects that, often after staying in prestigious research centers, have difficulty returning to their low–medium income countries of origin, to deposit and sow the seeds of their experience there.

## Figures and Tables

**Table 1:** Headache preparedness process.

Building the event and governance
Trained resources identification
Surveillance network action
Risk assessment evaluation
Event management
Post-event revision
Lesson learned analysis
